# New measures to assess the “Other” three pillars of food security–availability, utilization, and stability

**DOI:** 10.1186/s12966-023-01451-z

**Published:** 2023-04-26

**Authors:** Eric E. Calloway, Leah R. Carpenter, Tony Gargano, Julia L. Sharp, Amy L. Yaroch

**Affiliations:** 1 The Gretchen Swanson Center for Nutrition, 14301 FNB Parkway, Suite 100, Omaha, NE 68154 USA; 2 Colorado State University, Fort Collins, CO USA

**Keywords:** Food security, Availability, Utilization, Stability, Four pillars, Measurement development, Health food access barriers

## Abstract

**Background:**

In recent reviews of available measures, no existing measures assessed all four pillars of food security and most only assessed one or two pillars–predominantly the access pillar. The purpose of this study was to preliminarily develop novel measures of availability, utilization, and stability that are complementary to the USDA’s household food security survey measure (HFSSM).

**Methods:**

A formative phase included an expert advisory group, literature scans, and interviews with individuals experiencing food insecurity. From April-June 2021, the new measures were piloted in five states (California, Florida, Maryland, North Carolina, and Washington). The cross-sectional pilot survey included the new measures (perceived limited availability, utilization barriers, and food insecurity stability), scales and items for validation (e.g., food security, and self-reported dietary and health outcomes), and demographic questions. Exploratory factor analysis was used to assess dimensionality, internal consistency was assessed using Kuder-Richardson formula 21 (KR21), and convergent and discriminant validity were assessed using Spearman’s correlation coefficients. Also, a brief screener version was created for the utilization barriers measure that may be necessary for certain applications (e.g., clinical intake screening to inform referrals to assistance programs).

**Results:**

The analytic samples (perceived limited availability (n = 334); utilization barriers (n = 428); food insecurity stability (n = 445)) were around 45 years old on average, most households had children, over two-thirds were food insecure, over three-fourths were women, and the samples were racially/ethnically diverse. All items loaded highly and unambiguously to a factor (factor loadings range 0.525–0.903). Food insecurity stability showed a four-factor structure, utilization barriers showed a two-factor structure, and perceived limited availability showed a two-factor structure. KR21 metrics ranged from 0.72 to 0.84. Higher scores for the new measures were generally associated with increased food insecurity (rhos = 0.248–0.497), except for one of the food insecurity stability scores. Also, several of the measures were associated with statistically significantly worse health and dietary outcomes.

**Conclusions:**

The findings support the reliability and construct validity of these new measures within a largely low-income and food insecure sample of households in the United States. Following further testing, such as Confirmatory Factor Analysis in future samples, these measures may be used in various applications to promote a more comprehensive understanding of the food insecurity experience. Such work can help inform novel intervention approaches to address food insecurity more fully.

**Supplementary Information:**

The online version contains supplementary material available at 10.1186/s12966-023-01451-z.

## Introduction

The United States (U.S.) Department of Agriculture (USDA) defines food security as, “access by all people at all times to enough food for an active and healthy life.” The lack of food security (i.e., food insecurity) in the U.S. affects nearly one-in-eight households [1]. It is important to understand food insecurity since it is associated with increased risk for growth and cognitive development in children [2], parental aggravation and negative child behaviors [3], psychosocial stress [4], obesity [5], hypertension [6, 7], diabetes [7, 8], and chronic kidney disease [9]. The relationships between food insecurity and chronic diseases are seen even after controlling for socioeconomic factors [8]. Food insecurity is not merely a proxy measure for low socioeconomic status [10], but it exerts a direct influence on chronic disease risk, likely via compromises to dietary quality [11, 12] and trade-offs households make when allocating resources to food versus medical costs [13].

Food security in the U.S. is measured using the USDA Household Food Security Survey Module (HFSSM), which focuses primarily on perceived financial access to food [14–16]–aligned with USDA’s definition that focuses on *access* to food. The current widely held international definition of food security, put forth by the Food and Agriculture Organization (FAO) of the United Nations is that food security exists when “all people, at all times, have physical, social and economic access to sufficient, safe and nutritious food to meet their dietary needs and food preferences for an active and healthy life.” [17]. This is similar to USDA’s definition but adds further context to food insecurity by delving into types of access and types of food. Further, food security has traditionally been operationalized by the FAO as having four pillars–“availability,” “accessibility,” “utilization,” and “stability.” Also, recently, the emerging pillars of “agency” (“…the capacity of individuals. or groups to make their own decisions about what foods they eat, …produce, and how food is produced, processed and distributed…”) and “sustainability” (“…the long-term ability of food systems to provide food security and nutrition in a way that does not compromise… future generations.”) have been initially conceptualized by the FAO [18]. However, these emerging pillars were beyond the scope of this study.

The “other three pillars” (e.g., availability, utilization, and stability) are hierarchical [19] and emerged over time as food security was better understood [20]. Food security was originally conceptualized as an issue of availability, which is the physical presence of enough healthful food (e.g., fruits and vegetables) [19, 21, 22]. Sen et al., [23] studied historical records of famines and found that it is not enough for food to simply be physically present, households must also be able to access the food, such as having the economic and physical resources to acquire food [14, 19, 22]. Third is utilization, which refers to households’ ability to use food that they have access to safely prepare and store healthful meals [14, 19, 22]. Lastly, the stability pillar refers to the idea that availability, accessibility, and utilization can vary over time–being transitory, cyclical, or chronic [14, 19, 22].

While these international definitions and conceptualizations represent a broad view of food insecurity, measurement has primarily focused on a household’s *monetary* resources. The roots of food insecurity measurement were based originally in population-level econometric approaches that examined factors, such as agricultural production [23, 24]. Since the 1980s and 90s, the field has shifted to what are termed experience-based measurement approaches [24–26]. These are typically surveys that ask participants to answer questions about their subjective experience with food insecurity. The HFSSM is an example of an experience-based measure. While such tools are more practical for use by intervention implementers and program evaluators, compared to measures of agricultural production, for example, there is still room for improvement in terms of assessing a broader picture of food insecurity and closely related antecedents and barriers.

Since the late 1990s and early 2000s, academics and researchers in the U.S. have rallied around the HFSSM. This shared measurement across studies has led to an explosion in our understanding of the health consequences of food insecurity and disparities in food insecurity rates. The HFSSM has been widely used and rigorously tested [20, 27, 28]. However, by design, the HFSSM focuses primarily on one pillar, access, and specifically financial access (i.e., food affordability) by asking households about the experience of skipping meals and running out of food (or worrying about running out of food) due to not having enough money [14–16]. Further, in recent reviews of experience-based measures used globally, no existing measures assessed all four pillars of food security and most only assessed one or two pillars–predominantly the access pillar [14, 28]. Not having standard measures to assess the full breadth of the food insecurity experience can cause under-counting, such as households who can afford food but may not have food stores (e.g., grocery, supermarkets, corner stores, etc.) with healthful options nearby or lack equipment/space to cook healthful meals, or those who may experience food insecurity at only certain times of the year or month. Understanding these issues are crucial for monitoring food security more comprehensively and developing tailored intervention approaches.

The purpose of this study was to develop measures of availability, utilization, and stability that can be used complementary to the HFSSM, which already assesses accessibility. The items were worded similarly (i.e., all negatively worded), with the same response options (i.e., often true, sometimes true, and never true), the same recall period (i.e., 12 months), and can be scored in a similar manner (i.e., sum of affirmative responses). Therefore, if deemed reliable and valid, such measures could conceivably be used alongside the HFSSM in order to capture a comprehensive assessment of all four pillars of food security.

## Materials and methods

### Study overview

From January 2020–December 2021, the study authors sought to identify food-insecurity-related measurement gaps and develop measures to address those gaps. There were three measurement gaps addressed in the overall study, this paper reports on development and validation of measures to assess one of those gaps–the other three pillars of food security that are not emphasized in the HFSSM. More background on the overall study can be found elsewhere [29]. The work was completed in two main phases. First, a formative phase focused on identifying the measurement gap and developing item pools to address the gap. Second, a testing phase included administering a pilot survey and performing psychometric analyses. Exploratory factor analysis was performed and construct validity was assessed. Also, brief “screener” versions of the measures were identified for longer scales. Analyses were conducted using SAS version 9.4. The study application was reviewed by [University of Nebraska Medical Center] Institutional Review Board and the study authors were authorized to begin research. Interviewees provided oral informed consent and survey respondents provided written informed consent. All prevailing ethical standards in protecting human subjects were followed.

### Formative phase overview

We will describe the formative work briefly and refer the readers to Calloway et al. [29] for a more complete account. The purpose of the initial formative steps (January 2020–January 2021) was to create testing-ready pools of items that would be used for the testing phase. The inputs for this phase included an Expert Advisory Group (EAG), literature scans, and formative and cognitive interviews. The EAG (university researchers (n = 7), food insecurity non-profits leaders (n = 6), and federal government staff (n = 1)) helped prioritize measurement gaps and items, refine operational definitions, and provide advice on testing plans. Next, two iterative literature scans were conducted to identify and classify existing survey items that could be used and modified. Also, new items were created as needed. All candidate items were presented to the EAG. Following the literature scans, semi-structured 60-minute formative interviews (n = 47; 42 in English, 5 in Spanish) were conducted with adults experiencing food insecurity or at risk for food insecurity across five states (Arkansas, California, Maryland, Nebraska, and Tennessee) to understand their experiences and ensure the selected items were relevant. Items were then arranged into draft versions of the new measures – ready for cognitive interviewing. A total of ten cognitive interviews were conducted from December 2020 to January 2021 with adults (two men and eight women) experiencing food insecurity or at risk for food insecurity from Nebraska (n = 7) and California (n = 3). Interviews lasted approximately 60 min and employed a ‘think aloud’ technique in which participants explained their thought process while answering questions in the draft survey [30]. Revisions were made to make wording easily interpretable, reduce cognitive burden, and to prioritize or delete items.

### Testing phase

#### Piloting the Survey

Survey items resulting from the formative phase were tested in samples recruited with the help of different partner organizations than the formative phase across five states, California, Florida, Maryland, North Carolina, and Washington. A survey was created for pilot testing which included items for the new measures, scales and items needed for validation, and sociodemographic questions. Partner organizations (n = 7) that worked with households at risk for or experiencing food insecurity (e.g., food pantries, shelters, resource center, etc.) across the aforementioned states recruited survey participants from April to June 2021. Inclusion criteria were that the respondent was at least 18 years old, understood English, could answer questions about themselves and the household, and was from a household experiencing food insecurity or at risk for food insecurity. The partner organizations were asked to recruit a total of approximately 200 respondents per state, and sample diversity was monitored for race/ethnicity, age, gender, household composition (e.g., with/without children, single adults, cohabitating adults, etc.), rurality, and gradients of income across lower income levels with an aim to ensure sample diversity similar to populations the sites served. Recruitment and data collection procedures were tailored to each site. Recruitment occurred via email, texting, and/or flyer. In order to reach more of the populations served at each data collections site, sites advised the research team to offer both paper and web-based survey options and sites utilized these survey modes based on the needs of their participants. Test bias by survey mode was investigated in the psychometric analyses. Pilot surveys contained approximately 75–85 items each. One survey was completed per household and respondents received $25 gift cards for completing the survey.

#### Survey variables

In addition to the items for the new measures and sociodemographic questions, the following variables were included in the pilot survey and used in the analyses to assess convergent and discriminant validity.

##### Food insecurity

The USDA HFSSM, 18-item version, was used to assess household food security [31]. Households were assigned food security categories based on the number of affirmative (i.e., “Sometimes true” or “Often true”) responses (0 affirmative responses = “High food security;” 1–2 affirmative responses = “Marginal food security;” 3–7 for households with children or 3–5 for households without children = “Low food security;” 8–18 for households with children or 6–10 for household without children = “Very low food security”) [31]. These categories were treated as a four-level ordinal variable for the analyses, scored from 0 = “High food security” to 3 = “Very low food security” [31].

##### General health

Self-reported general health was assessed using an item from the Centers for Disease Control and Prevention’s (CDC) Behavioral Risk Factor Surveillance System (BRFSS) survey [32]. Respondents rated their general health from “Poor” (Scored as a 1) to “Excellent” (Scored as a 5). This was treated as a five-point ordinal score for the analyses.

##### Fruit and vegetable intake frequency

Items for whole fruits and vegetables (not including any potatoes or fruit juice) from the FRESH foods survey [33] were used to assess daily intake frequency of fruits and vegetables. Respondents were asked how frequently in the past 7 days they had consumed whole fruit, green salad, other vegetables, and beans. Response options were converted into daily frequencies for each of the four items and then summed to create a score for the fruit and vegetable food group. Those who indicated a daily fruit and vegetable intake frequency higher than three interquartile ranges above the median (i.e., above 8.75 times per day) were removed, which affected about 3% of the sample. This scoring process is based on a published scoring process for this survey administered within a similar sample [33].

##### Meal types

Items from the parent survey from the National Cancer Institute’s Food, Life, Activity, Sun, and Healthy Eating (FLASHE) study [34] were used to assess the frequency of consuming three different types of meals–fast food (i.e., a meal from a “fast food restaurant”), processed (i.e., a meal “made from a heat-and-serve package or box meal”), or scratch-cooked (i.e., a meal “cooked from scratch or a recipe”). The original items were modified by deemphasizing the timing (i.e., removed “evening meal”) and location (i.e., removed “at home”) in the questions and adding some examples discussed in the formative and cognitive interviews of foods and food outlets, where applicable. Response options asked on how many days in the past seven days they had each of the different types of meals and could range from 0 days to 7 days. The score from 0 to 7 for each meal types was used as an ordinal variable for the analyses.

##### Sports escapism

An item from a scale [35] assessing sports escapism (e.g., using sports as a past time to distract from usual day-to-day activities) was included to assess discriminant validity. The item chosen was, “Keeping up to date with sports provides an escape from my day-to-day activities” and response options were a seven-point Likert scale from “Strongly disagree” (scored as 1) to “Strongly agree” (Scored as 7). This item was modified to remove the original “Basketball” and replace it with “Keeping up to date with sports…” This item was chosen because it was not conceptually related to diet or moderators of diet (e.g., socioeconomic status) and was from a scale shown not to be associated with gender (and the item score was confirmed in this study not to differ significantly by gender). Responses to this question were treated as a seven-level ordinal variable for analyses.

#### Scoring the newly developed measures

The newly developed variables were made to be scored similarly to the HFSSM (i.e., summing affirmative responses). Like the HFSSM, where more affirmative responses indicate a greater degree of food insecurity, higher scores for the newly created measures indicate more limited food availability, more barriers to food utilization, and a greater degree of the four food insecurity stability types, respectively.

The abbreviation AvS and AvP refers to limited availability at stores and food banks/food pantries, respectively. U refers to utilization barriers items. C, S, M, and I refers to chronic, seasonal, intramonthly, and intermittent food insecurity stability, respectively. Items are numbered as well to give each a unique identifying code. The unique item codes and full item wording can be seen in the supplemental materials.

##### Perceived Limited Availability

Following the testing described in this study, there were three items assessing perceived limited availability at food stores (AvS1-3) and three items assessing perceived limited availability at food pantries (AvP1-3). For availability at stores and pantries, participants were asked about each location to assess if the “…food stores we went to…” or “…the places we got free food…” (these were also defined for the respondent), respectively, had very few “quality fruits and vegetables” (AvS1 and AvP1), “food we liked” (AvS2 and AvP2), and “foods that were good for our health and well-being” (AvS3 and AvP3). Only participants who indicated that they get food from food pantries were asked those three questions, and all participants were asked about perceived availability at food stores. Participants who selected “Sometimes true” or “Often true” were scored 1 for the item and those who select “Never true” were scored 0 for the item. Item scores were summed to create a 0–3 measure score for food stores and a 0–3 score for food pantries. See supplementary materials for full question wording and additional scoring guidance. The perceived limited availability sub-measures were considered separately in testing because they are responded to by different sub-samples (i.e., only those who used food pantries responded to the food pantry availability questions).

##### Utilization Barriers

The final eight items for the utilization barriers measure had response options of “Never true,” “Sometimes true,” and “Often true.” Participants who selected “Sometimes true” or “Often true” were scored 1 for the item and those who selected “Never true” were scored 0 for the item. Item scores were summed to create a 0–8 score. Items assessed having: safe storage for food (U1), cooking equipment (U2), other cooking utensils (U3), a sanitary food preparation area (U4), perceived knowledge for selecting healthful foods (U5), perceived “scratch-cooking” skills (U6), healthy cooking skills (U7), and time to prepare meals (U8). See supplementary materials for full question wording and additional scoring guidance. Utilization barriers was tested as one multidimensional measure.

##### Food Insecurity Stability

The scores to assess Food Insecurity Stability are calculated from three newly developed items that are *follow-ups* to three items in the existing HFSSM. These include HH2 (“(I/We) worried whether (my/our) food would run out before (I/we) got money to buy more.”), HH3 (“The food that (I/we) bought just didn’t last, and (I/we) didn’t have money to get more.”), and HH4 (“(I/we) couldn’t afford to eat balanced meals.”). These three items from the HFSSM comprise the “household” portion of the HFSSM [31]. These new follow-up items are anchored to HH2-HH4 of the HFSSM, and so they must be administered with the HFSSM (or at least with HH2-HH4). So, they can be thought of as a stability pillar supplement to the HFSSM. Also, because the HFSSM primarily assesses the access pillar, as noted in the introduction, the stability follow-ups that are anchored to it also primarily assess the *stability* of the access pillar.

The response options for the HFSSM questions are “never true,” “sometimes true,” or “often true.” If a participant selects “Sometimes true” to HH2-HH4, they are then asked one of the newly developed follow-up questions to clarify the timeframe that the statement is “Sometimes true” for their household (e.g., “In the last 12 months, when were you usually worried about running out of food?”).The options are (select all that apply): “Spring,” “Summer,” “Fall,” “Winter,” “Beginning of the month,” “Middle of the month,” “End of the month,” and “Randomly, no certain timeframe.” By selecting one or more seasons, a participant is assigned a score of one for seasonal food insecurity for that question. This can sum up to three if the participant selects one or more seasons for all three follow-up questions. By selecting one or more times of the month, a participant is assigned a score of one for intramonthly food insecurity for that question. This can sum up to three if the participant selects one or more times of the month for all three follow-up questions. By selecting “Randomly, no certain timeframe,” a participant is assigned a score of one for intermittent food insecurity for that question. This can sum up to three if the participant selects “Randomly, no certain timeframe” for all three follow-up questions. Finally, if the participant selects “Often true” for one of the HFSSM questions, they are not asked one of the newly developed follow-up questions but are assigned a score of one for chronic food insecurity for that question. This can sum up to three if the participant selects “Often true” for HH2, HH3, and HH4. Based on the summed scores, each participant should receive a score from 0 to 3 for chronic (C1-3), seasonal (S1-3), intramonthly (M1-3), and intermittent (I1-3) food insecurity. See supplementary materials for full question wording and additional scoring guidance. The food insecurity stability sub-scales were considered separately in testing because they measure different time components.

#### Data cleaning and assessing Missing Observation Percentages

A total of 517 surveys were at least 70% completed. Of these, 6 duplicate households were removed. “Speeders” (n = 17) who completed the survey too fast to be attentive (i.e., reading faster than 450 words per minute) and “straightliners/skippers” (n = 8) who skipped and/or selected only one of the response types for most of the survey items were removed [36]. There were 486 responses remaining for the initial assessment of missing observation percentage distributions. Also, items with excessive (≥ 15%) missing responses (i.e., a combination of skipped, “don’t know,” or “not applicable”) were removed. Participants that had complete data for the remaining candidate items were included in the analytic samples, which was utilized for the rest of the analyses (Food Insecurity Stability (n = 445); Utilization Barriers (n = 428); Perceived Limited Availability (n = 334).

#### Psychometric testing

Using unweighted least squares exploratory factor analysis with quartimin (oblique quartimax) rotation, items that did not load unambiguously (i.e., factor loading < 0.4) to one of the factors were removed [37]. A holistic assessment of scree plots, eigenvalues, and conceptual meaning were used to determine the number of factors to extract. Kuder-Richardson Formula 21 (KR21) (for binary items) was used to assess internal consistency of the measures/scales, with ≥ 0.70 used as an acceptable standard [38]. Lastly, analysis of variance using general linear models were used to examine potential test bias by assessing moderation of the relationship between the new measures’ scores and food security status by race, gender, age, education, and survey mode (i.e., online versus paper survey). Test bias was assessed by examining changes in the magnitude of the relationship between the new measures’ scores and a variable they are theoretically associated with (e.g., food security status). Statistically significant (p < 0.05) interaction terms indicated potential test bias [39].

#### Construct Validity Approach

Spearman’s rank correlation was used to assess convergent and discriminant validity by assessing statistical relationships between the new measures and previously used scales and survey items. It was hypothesized that the newly developed measures should each be negatively associated general health [32], fruit and vegetable intake frequency [33], and frequency of consuming “scratch-cooked” meals [34]. In addition, it was also hypothesized that the newly developed measures should each be positively associated with food insecurity [31], and the frequency of consuming “fast-food” and “processed/packaged” meals [34]. For discriminant validity, it was hypothesized that there should be no association with “sports escapism”[35] for either of the new measures. There were seven hypotheses examined for each of the seven new measures, therefore, the Bonferroni procedure was used to adjust the alpha level of statistical significance to 0.001 (i.e., 0.05/49) to limit the familywise error rate.

#### Determining brief Screener Version

Brief (e.g., one or two item) versions of the final measures may be necessary for certain applications. For example, for screening purposes (e.g., clinical intake screening to inform referrals to assistance programs) feasibility of administration may be more important than absolute accuracy, making longer measures often inappropriate. We assessed the screener versions based on their ability to categorize respondents as having a “High” score (i.e., above the sample median scores for the full measures). Desirable screening performance was high sensitivity and specificity, and inter-test reliability (Cohen’s kappa) of ≥ 0.6 [40]. There are not established standards for what constitutes high sensitivity and specificity, and necessary levels of sensitivity and specificity are very context specific (e.g., screening for life-threatening disease versus intake screening to inform assistance referrals). We chose 85% for sensitivity and 75% for specificity as desirable thresholds for the screeners. These thresholds allow for a precise screening (e.g., a high percentage of all “high” scoring households are correctly identified), but a relatively more moderate threshold for reliably identifying only “high” scoring households (e.g., some households screened as “high” are not “high” for the full measure). These tools will be used for screening households at risk to refer to programs or assistance, rather than medical procedures, and so false positives are not as important as false negatives in this context. For final measures that were four items long or longer (ultimately, only the utilization barriers scale met this criterion), all two-item combinations were assessed.

## Results

### Formative phase

The EAG prioritized three measurement gaps, one of which was the assessment of the “Other” Three Pillars. After reviewing the literature and preliminary formative interview findings, it was confirmed that measures were still needed to assess three of the four pillars of food insecurity that are not assessed by the HFSSM. Definitions were developed after reviewing the scientific literature and in consultation with the EAG. The literature was reviewed for existing relevant survey items and new items were created when needed. A total of 45 candidate items were reviewed by the EAG, with 22 ultimately moving on to cognitive interviews. This included 4 for perceived limited availability, 11 for utilization barriers, and 7 for food insecurity stability.

Over three rounds of cognitive interviews, items were modified based on interviewee recommendations. Modifications included wording changes for clarity, reducing cognitive burden, streamlining sentences, and modifying formatting. For example, the item, “In the last 12 months, we did not have pots, pans, a stirrer, can opener, knife, or other things needed to cook meals” was modified to, “In the last 12 months, we did not have the kitchen tools or utensils needed to cook meals (e.g., pots, pans, a stirrer, can opener, knife, spoons/forks, or other utensils).” This change prevented the respondent from having to read the list of kitchen utensils, instead, only the term “kitchen tools or utensils” is used and the examples are provided if needed. Interviewees also provided advice about items to cut or add and provided insight into how they interpreted questions that informed modifications. Examples of added items in response to interviewee recommendations included a ‘time barrier’ item to the utilization barriers measure and separating the perceived limited availability questions into two sets by places where food in purchased and places donated food is received for free. Interviewees reported thinking about these two types of locations very differently and separating the two made the questions easier to answer. See supplemental tables for final item wording. Following cognitive interviewing, the following were included in the pilot survey: 8 perceived limited availability (food store availability: AvS1-4; food pantry availability: AvP1-4), 8 utilization barriers (U1-8), and 3 food insecurity stability items from which scores for C1-3 (chronic), S1-3 (seasonal), M1-3 (intramonthly), and I1-3 (intermittent) were derived.

### Testing phase

#### Missing percentage and sample characteristics

Items AvS4 (i.e., limited foods available that met “religious or cultural needs” at food stores) and AvP4 (i.e., limited foods available that met “religious or cultural needs” at food pantries) had missing observation percentages > 40%, with many selecting “not applicable,” and were removed. All other items had percentages of missing observations < 5%. After removing the items with high missing observation percentages, the analytic samples for each measure for the remaining analyses included participants that had complete data for all the remaining candidate items (Food Insecurity Stability (n = 445); Utilization Barriers (n = 428); Perceived Limited Availability (n = 334)). Respondents were around 45 years old on average, most households had children, over two-thirds were food insecure, over three-fourths were women, and the sample was racially/ethnically diverse (Table 1). Approximately three-fourths completed their survey online and the remainder completed a paper survey. Those who were able to participate via paper surveys, compared to online surveys, were more likely to be men, be White (non-Hispanic), be above the sample median for age, and not have participated in post high school education (ps < 0.05).

**Table 1:** Selected sample characteristics for the analytic samples for each of the new measures

Sample Characteristics		Food Insecurity Stability (n = 445)	Utilization Barriers (n = 428)	Perceived Limited Availability (n = 334)
Age (years)	Mean (SD; Range)	44.8 (14.5; 18–86)	45.3 (14.6; 18–86)	44.8 (14.0; 18–81)
Proportion of federal poverty level	Mean (SD; Range)	0.73 (0.60; 0.05–4.89)	0.74 (0.56; 0.05–3.62)	0.77 (0.60; 0.05–3.62)
Daily fruit and vegetable intake frequency		2.03 (1.50; 0.00-7.29)	2.04 (1.52; 0.00-7.29)	2.09 (1.51; 0.00–7.00)
Weekly number of days eating a “scratch-cooked” meal		3.17 (2.33; 0.00–7.00)	3.31 (2.31; 0.00–7.00)	3.33 (2.33; 0.00–7.00)
Weekly number of days eating a “fast food” meal		0.95 (1.18; 0.00–7.00)	0.92 (1.16; 0.00–7.00)	0.94 (1.19; 0.00–7.00)
Weekly number of days eating a “processed” meal		1.82 (1.76; 0.00–7.00)	1.76 (1.74; 0.00–7.00)	1.92 (1.80; 0.00–7.00)
Food pantry utilization (%)		76%	75%	75%
Households with children (%)		59%	58%	60%
Women (%)		77%	79%	79%
Food Insecurity Status (%)	High	16%	19%	18%
	Marginal	12%	14%	13%
	Low	30%	29%	30%
	Very Low	41%	38%	39%
Reported General Health	Excellent	3%	2%	2%
	Very Good	10%	10%	10%
	Good	33%	33%	33%
	Fair	42%	43%	44%
	Poor	12%	12%	12%
Educational Attainment (%)	Less than high school	10%	8%	7%
	High school diploma or G.E.D.	35%	34%	31%
	Some college	26%	27%	28%
	Associates degree or greater	30%	31%	33%
Race or Ethnicity (%)	White, non-Hispanic	44%	45%	46%
	Latino/Hispanic	24%	23%	23%
	Black, non-Hispanic	17%	17%	18%
	Multi-racial/-ethnic, or another not listed	7%	8%	7%
	Asian, non-Hispanic	5%	5%	5%
	Tribal/Indigenous, non-Hispanic	2%	1%	1%
State	California	25%	25%	27%
	Florida	20%	20%	20%
	Maryland	16%	17%	16%
	North Carolina	19%	18%	19%
	Washington	20%	19%	18%

#### Psychometric Assessment

Exploratory factor analysis was conducted to examine the factor (F) structure of the candidate sets of items (Table 2). All items loaded highly and unambiguously to a factor. Food Insecurity Stability showed a four-factor structure, with items related to chronic food insecurity (F1: Eigenvalue = 2.15; KR21 = 0.79), intermittent food insecurity (F2: Eigenvalue = 1.97; KR21 = 0.77), seasonal food insecurity (F3: Eigenvalue = 1.28; KR21 = 0.76), and intramonth food insecurity (F4: Eigenvalue = 0.90; KR21 = 0.72). Utilization barriers showed a two-factor structure, with items related to tangible barriers (e.g., safe storage for food, cooking equipment, other cooking utensils, and a sanitary food preparation area) (F1: Eigenvalue = 3.35; KR21 = 0.85) and intangible barriers (e.g., perceived knowledge for selecting healthful foods, perceived “scratch-cooking” skills, healthy cooking skills, and time to prepare meals) (F2: Eigenvalue = 1.05; KR21 = 0.79). The KR21 for all eight items in the utilization barriers measure was 0.84. Perceived limited availability showed a two-factor structure, with items related to pantry availability (F1: Eigenvalue = 2.40; KR21 = 0.78) and store availability (F2: Eigenvalue = 0.78; KR21 = 0.76).

**Table 2:** Factor loadings from exploratory factor analysis findings showing the factor structures for the new measures

Food Insecurity Stability (n = 445)					Utilization Barriers (n = 428)			Perceived Limited Availability (n = 334)		
Item	F1	F2	F3	F4	Item	F1	F2	Item	F1	F2
C2	**0.869**				U2	**0.896**		AvP1	**0.778**	
C3	**0.734**				U1	**0.792**		AvP3	**0.715**	
C1	**0.649**				U4	**0.682**		AvP2	**0.714**	
I2		**0.762**			U3	**0.658**		AvS2		**0.903**
I3		**0.727**			U6		**0.814**	AvS3		**0.640**
I1		**0.686**			U5		**0.764**	AvS1		**0.569**
S2			**0.740**		U7		**0.642**			
S3			**0.737**		U8		**0.525**			
S1			**0.676**							
M2				**0.791**						
M3				**0.662**						
M1				**0.604**						

F = Factor

C = items assessing chronic food insecurity

I = items assessing intermittent food insecurity

S = items assessing seasonal food insecurity

M = items assessing intramonth food insecurity

U = items assessing utilization barriers

AvP = items assessing perceived limited availability at food pantries

AvS = items assessing perceived limited availability at food stores

For the measure scores (Table 3**)**, respondents scored numerically higher for chronic food insecurity (mean = 0.74, SD = 1.09) and intramonth food insecurity (mean = 0.80, SD = 1.06), relative to seasonal (mean = 0.51, SD = 0.93) and intermittent food insecurity (mean = 0.56, SD = 0.96), and so they may experience more chronic food insecurity or intramonthly cycles of food insecurity, on average, compared to seasonal or intermittent variations in food insecurity. The utilization barriers score was on the lower end of the possible range (mean = 2.31, SD = 2.34; maximum score is 8), indicating that on average, respondents may encounter approximately two barriers to preparing healthy meals from the foods they have access to. Scores for the perceived limited availability at stores (mean = 1.69, SD = 1.21) were lower than for pantries (mean = 2.14, SD = 1.12), which is expected as food stores generally have a wider selection than food pantries. All KR21 scores indicated acceptable internal consistency for all measures (range = 0.72–0.84).

The association between perceived limited availability at pantries and food insecurity was moderated by educational attainment, indicating potential test bias. Therefore, in future applications of the perceived limited availability measure within samples from diverse educational backgrounds, the influence of education on the analyses should be assessed and controlled for if needed.

**Table 3:** Measure descriptions and psychometric findings for the newly created measures

	Food Insecurity Stability (n = 445)				Utilization Barriers (n = 428)	Perceived Limited Availability	
	Chronic	Seasonal	Intramonthly	Intermittent		Store (n = 334)	Pantry (n = 249)
*Measure Description*							
Number of items (Possible score range)	3 (0–3)	3 (0–3)	3 (0–3)	3 (0–3)	8 (0–8)	3 (0–3)	3 (0–3)
Measure mean score (sum score; Mean (SD), Median (IQR))	0.74 (1.09), 0.00 (0.00–1.00)	0.51 (0.93), 0.00 (0.00–1.00)	0.80 (1.06), 0.00 (0.00–2.00)	0.56 (0.96), 0.00 (0.00–1.00)	2.31 (2.34), 2.00 (0.00–4.00)	1.69 (1.21), 2.00 (1.00–3.00)	2.14 (1.12), 3.00 (1.00–3.00)
***Reliability and Test Bias***							
KR21	0.79	0.76	0.72	0.77	0.84	0.76	0.78
Moderation by educational attainment, age, race/ethnicity, gender, or test mode	None	None	None	None	None	None	Education

#### Convergent and discriminant validity

The Spearman’s correlation coefficients between the new measures and the validation variables indicated associations were largely in the expected directions (Table 4). Besides intermittent food insecurity, higher scores for all other measures/sub-scales in Table 4 were significantly associated with food insecurity. Utilization barriers and chronic food insecurity were significantly associated with poorer general health. For the dietary variables, chronic food insecurity, utilization barriers, and perceived limited availability at stores were associated with consuming fruits and vegetables and “scratch” cooked meals less frequently. Also, higher scores for chronic food insecurity and utilization barriers were associated with consuming processed meals more frequently. Frequency of fast-food intake was not significantly correlated with the new measures. Seasonal, intramonth, and intermittent food insecurity, as well as perceived limited availability at pantries had noticeably fewer significant associations and smaller effect sizes compared to the other measures/sub-scales. Finally, as hypothesized, there was no association between sports escapism and the new measures.

**Table 4:** Spearman’s correlation coefficients for assessing convergent and discriminant validity of the new measures

	Food Insecurity^A^	General Health	Fruits and Vegetables	Scratch Meals	Fast Food Meals	Processed Meals	Sports Escapism
Chronic (n = 445)	0.497*	-0.162*	-0.182*	-0.195*	-0.061	0.164*	-0.014
Seasonal (n = 445)	0.269*	-0.029	-0.114	-0.092	-0.001	-0.063	0.042
Intramonth (n = 445)	0.257*	-0.042	-0.058	-0.023	-0.016	0.009	0.077
Intermittent (n = 445)	0.134	-0.039	-0.084	-0.094	0.001	0.075	0.006
Utilization Barriers (n = 428)	0.484*	-0.195*	-0.258*	-0.273*	0.015	0.177*	-0.006
Perceived Limited Availability, Stores (n = 334)	0.342*	-0.167	-0.188*	-0.265*	0.106	0.166	0.081
Perceived Limited Availability, Pantries (n = 249)	0.248*	-0.099	-0.052	-0.017	0.144	0.113	0.118

A: Scored as a 4-point ordinal variable based on increasing categories of food insecurity (i.e., 0 = “High food security” to 3 = “Very low food security”)

* = Statistically significant at the Bonferroni adjusted 0.001 alpha level (i.e., p < 0.001)

#### Determining brief screener versions

A brief screener version was only considered for the utilization barriers measure because the other measures were already short, only requiring three items. To screen for “high” utilization barriers (i.e., above the sample median), all two-item combinations of the final eight items were assessed. Many combinations performed well as screeners, and the final selection was based on preference for higher sensitivity and having an item on each of the sub-scales. Defining a positive screen as responding “Sometimes true” or “Often true” to either U4 (a sanitary food preparation area) or U7 (healthy cooking skills) had 96% sensitivity, 81% specificity, and had a Cohen’s kappa of 0.727 for categorizing participants as facing a “high” degree of utilization barriers compared to the full measure. The screener scored well within desired thresholds for sensitivity, specificity, and kappa agreement scores and may be useful for applications where administering the full measure is not feasible (e.g., intake screening to inform program/assistance referral).

## Discussion and conclusion

Three new self-administered measures were created to assess three of the four pillars of food insecurity that are not currently emphasized with the HFSSM. The findings support the reliability and construct validity of these new measures within a largely low-income and food insecure sample of households in the U.S. However, further examination in future samples is warranted. In this study, higher scores indicated a greater degree of various types of food security instability, utilization barriers, and limited food availability. Generally, higher scores were associated with decreased food security. Also, several of the measures were associated with statistically significantly worse health and dietary outcomes. Finally, a brief two-item screener that showed a high degree of agreement with the full eight-item utilization barriers measure was identified. This may add to the measure’s feasibility in applications where brief versions are needed such as clinical intake screening.

The two “limited availability” measures were made up of three items each that assessed perceived limited availability of “quality fruits and vegetables,” foods good for “health and well-being,” and “food that we liked” at food stores and food pantries, respectively. The physical presence of food in an area can be measured objectively in many cases, such as using secondary data sources (e.g., concentration of “food outlets” in an area [41]) or environmental assessments (e.g., Nutrition Environment Measures Survey in Stores (NEMS-S) [42]). However, understanding perceived availability is still important. For example, secondary data might not be available or have enough granularity for a given study and collecting NEMS-S data may not always be feasible. Also, it can be difficult to determine which food sources are relevant to a community, as households may not be homogenous (e.g., they may not necessarily choose the nearest full-service grocery stores) [43]. Perceived availability collected from self-report survey items can be feasible and has been shown to have acceptable agreement with objective measures [44, 45]. Further, assessing perceived availability can allow for intervention tailoring to a specific sub-population within a geographic area and understanding availability within the locations that are familiar and relevant to the sub-population of interest. While there are existing perceived food availability measures [42, 45], the measures in the current study were the first to be developed to complement the HFSSM by adopting the wording style, response options, recall period, and scoring approach. Further, these new measures are unique in that they assess availability in two distinct environments (i.e., food retail and food pantries) relevant to food insecure populations and have questions assessing specific healthful foods, general healthful foods, and foods that meet respondents’ dietary preferences. Also, the new measures do not impose a geographic boundary, but instead ask respondents about availability among the places where they get food.

Perceived limited availability at food stores had stronger associations (e.g., correlations) with food security and dietary outcomes compared to food pantry availability in this study. For most food insecure households, food stores as opposed to food pantries provide most of the food to households [46]. So, it makes sense that perceived food store availability had a larger effect on dietary outcomes. Availability and concentration of stores with healthful food options has been shown in several studies to have a positive association with healthful dietary intake [47–52]. However, understanding perceived pantry availability is also important as research has shown that many households rely on food pantries for extended periods of time [53, 54] and that, on average, food pantry clients need support maintaining a healthful diet [55]. Future research can investigate differences in effect sizes on dietary outcomes among food insecure populations based on the proportion of food they obtain from food pantries.

The utilization barriers measure contained eight items that assessed tangible (e.g., not having food preparation equipment) and intangible (e.g., not knowing how to cook meals from “scratch”) barriers to being able to utilize food that a household has access to in order to prepare healthful meals. Having more barriers was associated with a greater degree of food insecurity, poorer health, and less healthful dietary outcomes. Studies have similarly found that tangible barriers such as lacking food preparation equipment, safe food storage, or kitchen facilities [56–59] and intangible barriers such as limited cooking self-efficacy, lack of cooking and food skills, and time constraints [52, 58, 60–66] were associated with food insecurity and/or less healthful dietary outcomes (e.g., consuming fewer fruits and vegetables). While there are existing self-administered measures to assess many of these variables [67–69], none have been developed in the U.S. to specifically assess the utilization pillar [14, 28], both tangible and intangible aspects, and to complement the HFSSM.

Stability measurement involved three items that are follow-up questions for respondents who selected “sometimes true” to HH2, HH3, and HH4 from the HFSSM. Based on responses, respondents get a score for their degree of chronic, seasonal, intramonthly, and intermittent food security. It is important to note that this refers to stability of the access pillar only, which is the primary pillar assessed by the HFSSM, while conceptually stability can be relevant to the availability and utilization pillars as well [19]. Future work will be needed to create assessments of stability for those other pillars. In this study, higher scores for chronic, seasonal, and intramonthly stability were each associated with more severe food insecurity measured using the HFSSM. Chronic food insecurity was also associated with poorer health and less healthful dietary outcomes. However, intermittent food insecurity was not significantly associated with the variables assessed. It is possible that this group experiences “low-level” food insecurity associated with rare one-off events (e.g., an unexpected bill) that they may have difficulty handling. Also, while intermittent food insecurity was not associated with increased food insecurity as measured by the HFSSM, experiencing intermittent food insecurity may be an early signal that a household is at risk for food insecurity (e.g., financial instability). More research is needed to explore this sub-group and the implications of being intermittently food insecure.

There has been limited research studying the stability pillar, possibly due to a lack of measures [14, 28], few longitudinal studies [70] and inconsistency in defining appropriate stability timeframes [20]. Nord [71, 72] described methods for using responses to the HFSSM to classify households as having “frequent or persistent” or “occasional or episodic” food insecurity. Among food insecure households, most only experienced “occasional or episodic” food insecurity, while the remaining 22% were “frequently or persistently” food insecure [71]. Other studies have shown similar findings among food insecure households [1, 73]. Individuals in households that are chronically food insecure and/or reliant on food pantries (e.g., frequent use for two years or more), compared to those that are not, have been shown to be more likely to be dependent on multiple forms of governmental assistance, have unmet physical and mental medical needs, be disabled, and have very low food security status [53, 54, 72, 74]. While confirming the length of chronic food insecurity was beyond the scope of this study, similar associations with health and severity of food insecurity were observed.

The current approaches utilizing the HFSSM can classify households by intermittent or chronic food insecurity, but they cannot assess seasonality or intramonth cycles. Seasonal food insecurity may be spurred by unreliable or inconsistent employment (e.g., seasonal employment in agriculture or shift work in hospitality sectors) [20], [75, 76]. These types of jobs may have busy and slow periods, based on seasonal cycles, such as harvesting and tourism. Further, research has shown seasonal food insecurity to be associated with seasonal fluctuations in household heating and cooling costs, especially among older adults, [77] and differences across states depending on the robustness of their Summer Food Service Program implementation among households with school-age children [78]. Intramonth food insecurity cycles have been studied in the context of the “food stamp cycle” [79]. Households who receive benefits from the Supplemental Nutrition Assistance Program (SNAP) tend to utilize their monthly benefits early in the month and then run out of food later in the month. This cycle is associated with monthly fluctuations in caloric intake, dietary healthfulness, difficulty managing chronic diseases, and reliance on food pantries towards the end of the month [79–84]. The newly created measures could be beneficial for researchers studying seasonal and intramonthly food insecurity and program implementers and evaluators working with household experiencing these issues.

### Study Limitations and Strengths

The findings should be interpreted in the context of the study limitations. Firstly, this study presents a hypothesized scale based on an exploratory factor analysis. Additional work in future samples should be conducted to confirm the findings are generalizable beyond this sample. Further, this study utilized a convenience sample and may not be representative of food insecure households or households at risk for food insecurity in the U.S. The survey was offered in both online and paper formats, and respondents differed demographically by which mode they utilized. Allowing the paper survey, in addition to the online survey, may have reduced sampling error (e.g., allowing more of the target population to participate who did not have access to the internet), but may have increased measurement error (e.g., differences in interface between the modes and lack of automated skip logic for paper surveys) [85, 86]. The rural U.S. was not well represented in this study. While there was some rural representation in parts of WA and NC, the sample generally skewed urban with recruitment sites located in Tampa Bay (FL), San Diego (CA), Seattle (WA), and the Washington D.C. metro area. More research is needed to investigate potential differences in rural versus urban contexts in the U.S. Also, men were not well represented in the sample. Research has shown that men and women in the same households interpret and respond differently to the HFSSM questions [87, 88] and investigating potential gender differences for these new measures is needed. Also, this study is not longitudinal, and therefore cannot assess agreement between observed and reported stability of food insecurity. Further, we do not have observed or objective measures of food availability at food locations relevant to the respondents, food preparation equipment in the households, or cooking skills. However, associations with measures of food security, health, and diet as assessed through correlations were in the expected directions based on the literature.

The strengths of this study are notable and include a robust formative phase incorporating evidence from the literature, experts, and individuals facing food insecurity, and comparison against validated and relevant measures to assess validity. Further, the sample was relatively large and diverse such as by educational attainment, age, race/ethnicity, and states across the U.S.

### Conclusions

This study described the preliminary development of a new suite of measures that complement the HFSSM to help capture all four pillars of food security (e.g., availability, accessibility, utilization, and stability). When used in conjunction with the HFSSM, these novel tools may help address a common critique of the HFSSM–that it only assesses the access pillar. However, more work is needed to identify best practices for administering the suite of measures together with the HFSSM. Also, the measures can be used separately if desired based on specific research or measurement objectives. As noted above, there is relatively limited research conducted on the “other” three pillars (that are not access), especially the stability pillar. Next steps for this work include disseminating these preliminary measures for others to confirm these findings in different samples, such as through confirmatory factor analysis and item response theory approaches. Pending further testing, these tools have the potential to offer a way for researchers to further explore these areas and understand the experiences of affected households. Further, the tools can be used to glean practical information. For example, assessing high scores on items within the utilization barriers measure can inform the development of intervention approaches and needs assessment. Much of the work to address food insecurity is conducted by non-profit food banks, food pantries, and community-based organizations who often operate on limited funding. The new measures are easily administered and scored by simply summing affirmative responses (without the need for advanced software) making them accessible for organizations that may have limited resources. We also envision organizations implementing these tools to include non-profit hospitals (e.g., Community Health Needs Assessment), philanthropic organizations (e.g., collective impact assessment), and other social service organizations (e.g., intake screening for service referral). These new tools can aid in developing a more comprehensive understanding of the food insecurity experience to better understand previously understudied food insecure sub-groups. Such work can help identify and develop novel intervention approaches to more fully address food insecurity.

## Electronic supplementary material

Below is the link to the electronic supplementary material.

Supplementary Material 1: Measures to Assess the Availability, Utilization, and Stability Pillars of Food Security: Scoring and Interpretation Guide

## Data Availability

The final measures and a detailed scoring guide can be found at our website (https://www.centerfornutrition.org/food-insecurity-measures). Also, the final items are included in the supplementary materials. The datasets generated and/or analyzed during the current study are not currently publicly available due to planned manuscripts but may be released publicly in the future. However, data may be made available from the corresponding author on reasonable request.

## Abbreviations

AvP: items assessing perceived limited availability at food pantries
AvS: items assessing perceived limited availability at food stores
BRFSS: Behavioral Risk Factor Surveillance System
C: items assessing chronic food insecurity
CDC: Centers for Disease Control and Prevention
EAG: Expert Advisory Group
F: Factor
FAO: Food and Agriculture Organization
FLASHE: Food, Life, Activity, Sun, and Healthy Eating
HFSSM: household food security survey measure
I: items assessing intermittent food insecurity
KR21: Kuder-Richardson formula 21
M: items assessing intramonth food insecurity
S: items assessing seasonal food insecurity
U: items assessing utilization barriers
U.S.: United States
USDA: United States Department of Agriculture
