# Supplementary material for: New measures to assess the “Other” three pillars of food security–availability, utilization, and stability
Source: Int J Behav Nutr Phys Act. 2023 Apr 26;20:51. doi: 10.1186/s12966-023-01451-z (PMC10134599; doi:10.1186/s12966-023-01451-z)
Supplement: Supplementary file 1 — Supplementary Material 1: Measures to Assess the Availability, Utilization, and Stability Pillars of Food Security: Scoring and Interpretation Guide [file 12966_2023_1451_MOESM1_ESM.pdf]

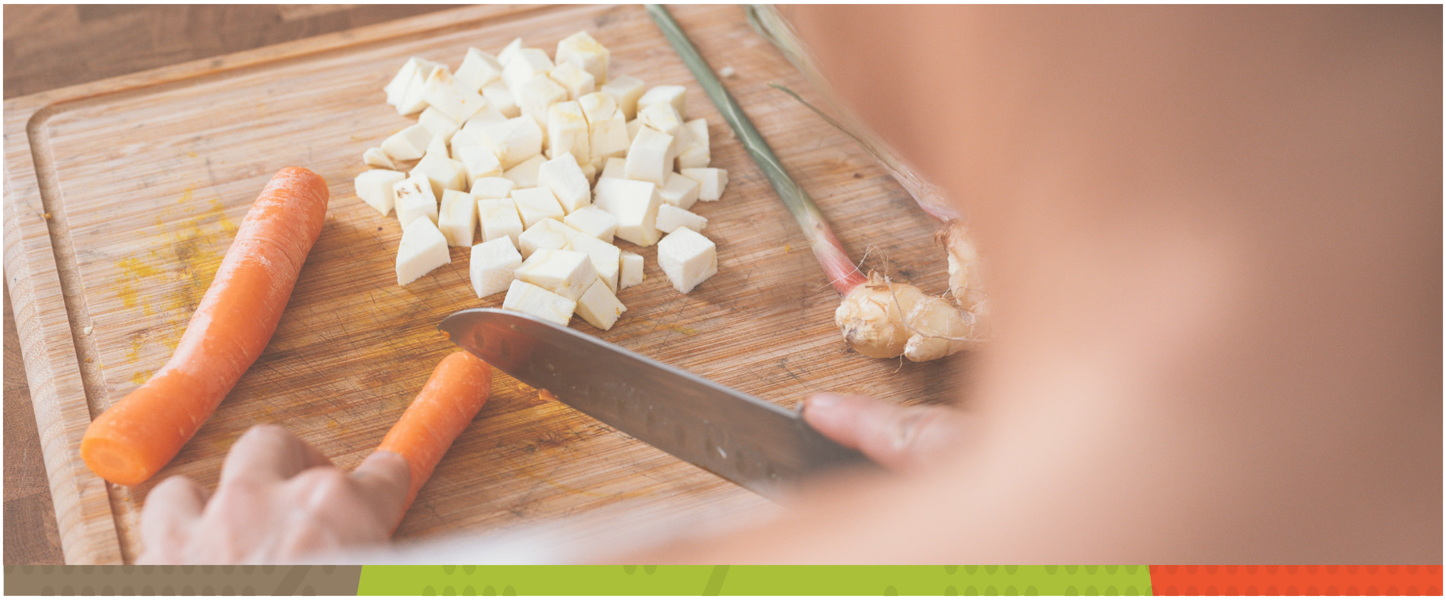

GRETCHEN SWANSON
CENTER FOR NUTRITION

RESEARCH ⋆ PARTNERSHIP ⋆ EVALUATION

Measures to Assess the Availability, Utilization, and Stability Pillars of Food Security

Scoring and Interpretation Guide

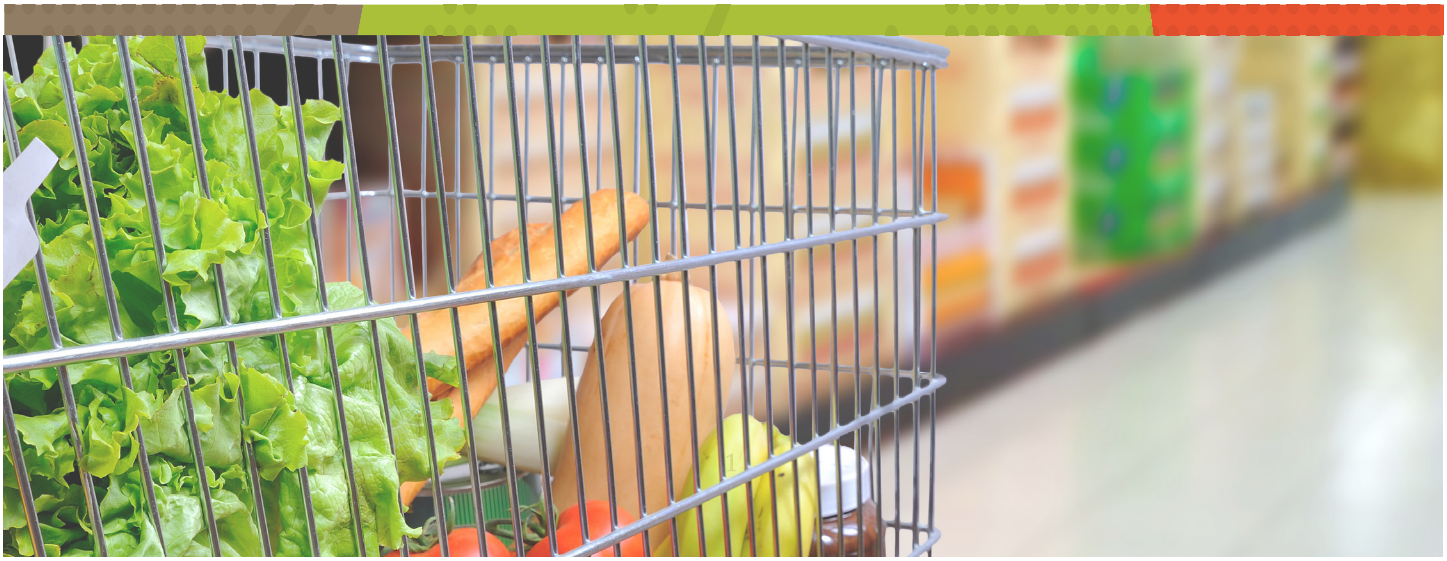

GRETCHEN SWANSON
CENTER FOR
NUTRITION

RESEARCH * PARTNERSHIP * EVALUATION

The Gretchen Swanson Center for Nutrition

The Gretchen Swanson Center for Nutrition (GSCN) is a non-profit research and evaluation organization providing scientific expertise and partnership in the key public health areas of healthy eating-active living, food insecurity, policy advocacy and health equity. GSCN specializes in the development and implementation of mixed-methods approaches, which are primarily focused on measuring changes that occur through policy, systems, and environmental interventions. GSCN works nationally providing research and evaluation services to communities, non-profits, academic and government institutions, and policy makers. For more information about the Center, please visit www.centerfornutrition.org.

Corresponding Author: Eric Calloway, RD, PhD; ecalloway@centerfornutrition.org
Supporting Authors: Leah Carpenter, MPH, Tony Gargano, MPH, Julia Sharp, PhD, and Amy Yaroch, PhD

Funding statement: The Development of this guide and the measures was made possible through funding provided by the Walmart Foundation.

Acknowledgments: Researchers at GSCN would like to thank members of the Food Insecurity Measurement Expert Advisory Group for their thoughtful input and guidance during the development of these measures: Dr. Heidi Blanck (Centers for Disease Control and Prevention), Heather Bruskin (Montgomery County Food Council), Dr. Uriyoan Colon-Ramos (George Washington University), Dr. Justin Denney (Washington State University), Emily Engelhard (Feeding America), Dr. Elizabeth Jimenez (University of New Mexico), Dr. Kathy Krey (Baylor University), Dr. Christopher Long (University of Arkansas for Medical Sciences), Dr. Katie Martin (Connecticut Foodshare), Dr. Angela Odoms-Young (Cornell University), Caitlyn Peacock (Tampa Bay Network to End Hunger), Dr. Hilary Seligman (University of California San Francisco), Dr. Shreela Sharma (University of Texas), and Reginald Young (Alameda County Community Food Bank).

We would also like thank the organizations that helped recruit interviewees and/or to pilot the measures across five states: Aaron's Place (MD), Cherokee Health (TN), Food Bank for the Heartland (NE), Gaithersburg HELP (MD), HopeSource (WA), Hunger and Health Coalition (NC), Manna Food Market (MD), Montgomery County DHHS (MD), Montgomery County Food Policy Council (MD), North County Food Pantry (WA), Nourish Now (MD), Tampa Bay Network to End Hunger (FL), Together (NE), UC San Diego - Center for Community Health (CA), University District Food Bank (WA), and University of Arkansas for Medical Sciences (UAMS) (AR).

GRETCHEN SWANSON CENTER FOR NUTRITION

RESEARCH * PARTNERSHIP * EVALUATION

Table of Contents

1. Introduction and Measures Description 4
2. Potential Uses of the Measures 5
3. Scoring and Interpreting the Measures 6
  3.1. Perceived Limited Availability Scoring and Interpretation 6
  3.2. Utilization Barriers Scoring and Interpretation 7
  3.3. Food Insecurity Stability Scoring and Interpretation 8
Appendix 10

1. Introduction and Measures Description

This User's Guide provides instruction for scoring and interpreting three measures to assess Perceived Limited Availability, Utilization Barriers, and Food Insecurity Stability. Like the United States Department of Agriculture's (USDA) household food security survey module, where higher scores indicate a greater degree of food insecurity, higher scores for the measures described in this guide indicate more limited availability, more barriers to utilization, and a greater degree of four food insecurity stability types, respectively. Therefore, higher scores are considered less desirable.

The guide provides a brief background on the development of the measures, descriptions of the measures, examples for potential uses, guidance for scoring and interpreting scores. The measures and supporting materials and resources can be found on our website. The measures can be used freely and without permission from the authors as long they are implemented according to guidance provided in this user's guide and/or the peer-reviewed study describing the development and validation of these measures, also found on our website, and have cited the peer-reviewed study in any publications developed utilizing these measures.

The items were developed in an iterative process involving input from the scientific literature, an expert advisory group, and interviews with individuals that have experienced or were at risk for food insecurity. The developed items then underwent a cognitive interviewing process where clarity was assessed and wording was refined. Next, the items were pilot tested in a multi-state sample and underwent psychometric testing and validity assessment. For a detailed description of the methods used for developing and validating these measures, please read the corresponding peer-reviewed study mentioned above.

Table 1, below, shows a description of the three measures, along with information about item counts, descriptive statistics, and internal consistency of the measures. These measures are modular and can be used separately or as a set based on the objectives and interests of those implementing them. **To view the tools in other languages, visit www.centerfornutrition.org/food-insecurity-measures/other-3-pillars.**

Table 1. Descriptions of the Availability, Utilization, and Stability Measures

Measure	Description	Item Count	Score range	Mean Score (SD)[A]	Median (IQR)[A]	Cronbach's Alpha[A]
Perceived Limited Availability	Assesses perceived availability of fruits and vegetables, healthful food, and liked foods at food stores and at food pantries.	Stores: 3	0-3	1.69 (1.21)	2.00 (1.00-3.00)	0.76
		Pantries: 3	0-3	2.14 (1.12)	3.00 (1.00-3.00)	0.78
Utilization Barriers	Assesses households' barriers to being able to use food that they have access to in order to produce healthful meals.	8	0-8	2.31 (2.34)	2.00 (0.00-4.00)	0.84
Food Insecurity Stability	Assesses the temporal state of a household's food insecurity – chronic, seasonal, intra-monthly, and intermittent food insecurity.	Chronic: 3[B]	0-3	0.74 (1.09)	0.00 (0.00-1.00)	0.79
		Seasonal: 3[B]	0-3	0.51 (0.93)	0.00 (0.00-1.00)	0.76
		Monthly: 3[B]	0-3	0.80 (1.06)	0.00 (0.00-2.00)	0.72
		Intermittent: 3[B]	0-3	0.56 (0.96)	0.00 (0.00-1.00)	0.77

[A]Descriptive statistics and Cronbach's alpha based on data from a largely low-income and food insecure sample from CA, FL, MD, NC, and WA
[B]3 total items are used to create scores for each of the four food insecurity stability types

2. Potential Uses of the Measures

The measures can be used in a variety of ways, such as needs assessments, program evaluations, clinical screenings and other research activities. The following table presents these uses along with descriptions and examples to further illustrate their potential use. These measures are modular and can be used separately or as a set of two or three measures depending on the needs of the project. For more examples of potential uses, visit https://www.centerfornutrition.org/food-insecurity-measures

Table 2. Potential Uses Across Community and Clinical Settings

Potential Uses	Description	Example Types of Organizations	Example Projects
Needs Assessment	Identifies key health needs and issues through systematic, comprehensive data collection and analysis.	• Anti-hunger non-profits/non-governmental organizations<br>• Health Departments<br>• Non-Profit Hospitals	A non-profit hospital working with their community could utilize the measures as part of their Community Health Needs Assessment process.
Program Evaluation	Program evaluation is a systematic way to improve and account for public health actions by involving procedures that are useful, feasible, ethical, and accurate.	• Anti-hunger non-profits/non-governmental organizations<br>• Health Department<br>• Philanthropic organizations	An anti-hunger non-profit conducting community programming could utilize the measures as part of their program evaluation to assess impacts.
Intake/Clinical Screening	Screening refers to the use of brief measures to assess risk and identify individuals in need of additional support and/or resources.	• Hospitals/Clinics<br>• Social services (e.g., WIC clinics)<br>• Food pantries/food banks	A food pantry could utilize the brief screener version(s) of the measures as part of their client intake process to screen for households at risk and inform food distributions and referrals to services.
Research/Surveillance	Public health surveillance is the ongoing, systematic collection, analysis, and interpretation of health-related data essential to planning, implementation, and evaluation of public health practice.	• Researchers<br>• Governmental agencies	The measures could be added to an existing surveillance system conducted by a government agency aimed at measuring factors related to food insecurity, to see trends in the measures over time.

3. Scoring and Interpreting the Measures

3.1. Perceived Limited Availability

Table 3a & 3b shows the six items for the Perceived Limited Availability measure. There are three items assessing perceived limited availability at food stores (AvS1-3) and three items assessing perceived limited availability at food pantries (AvP1-3). The types of food include “quality fruits and vegetables,” “food we liked,” and “foods that were good for our health and well-being.” Only participants who indicated that they get food from food pantries were asked those three questions (AvP1-3), and all participants were asked about perceived availability at food stores. To ensure respondents who use food pantries are also asked AvP1-3, a question needs to be included in the survey that asks about their use of food pantries (See the **Appendix** for items A & B which can be used to assess food sources in general and also food pantry use to inform survey skip patterns). Participants who selected “Sometimes true” or “Often true” were scored 1 for the item and those who select “Never true” were scored 0 for the item. Item scores were summed to create a 0-3 measure score for food stores and a 0-3 score for food pantries. Higher scores are considered less desirable.

Households that score higher perceive there is lower availability of healthful foods and foods that meet their preferences at the locations where they source food. In a large sample of racially/ethnically diverse and largely low-income and food-insecure households across five states (CA, FL, MD, NC, and WA), “High” scores for both measures were 3.0, which was at or above the sample median and the maximum score. Higher scores for both store and pantry availability were associated with being food insecure, and higher scores for store availability were associated with less frequent consumption of fruits and vegetables and “scratch-cooked” meals.

In addition to determining “low” and “high” scores, there may also be a need to understand how “high” scores could be improved. This measure offers practical information, particularly when combined with items A and B (see **Appendix**), that allow respondents to indicate their food sources. This information can help identify communities with limited food availability to inform policy, systems, and environmental interventions. Also, food banks and food pantries could use the pantry availability items to monitor the degree to which their clients perceive healthful foods and preferred foods are made available.

The potential for test bias was examined by test mode (paper versus web-based), age, race, gender, and education. There was potential test bias detected by educational attainment in the pilot study sample for the perceived limited availability at pantries measure. Therefore, in future applications of the perceived limited availability measure within samples from diverse educational backgrounds, the influence of education on the analyses should be assessed and controlled for if needed. No other test bias was detected.

Table 3a. Items comprising the Food Store Perceived Limited Availability measure along with item scoring and measure scoring

Item ID	Question Text	Response Options to All Items	Item Score	Measure Score
AvS1	In the last 12 months, the food stores (I/we) went to had very few quality fruits and vegetables.	Never True = 0<br>Sometimes True = 1<br>Often True = 1		Sum of Item Scores
AvS2	In the last 12 months, the food stores (I/we) went to had very few foods that (I/we) liked.			
AvS3	In the last 12 months, we worried that the food we were able to eat would hurt our health and well-being.			

Table 3b. Items comprising the Food Pantry Perceived Limited Availability measure along with item scoring and measure scoring

Item ID	Question Text	Response Options to All Items	Item Score	Measure Score
AvP1	In the last 12 months, the places (I/we) got free food had very few quality fruits and vegetables.		Never True = 0<br>Sometimes True = 1<br>Often True = 1	Sum of Item Scores
AvP2	In the last 12 months, the places (I/we) got free food had very few foods that (I/we) liked.			
AvP3	In the last 12 months, the places (I/we) got free food had very few foods that were good for (my/our) health and well-being.			

3.2. Utilization Barriers

Table 4 shows the eight items for the Utilization Barriers measure. There are four items that assess tangible barriers (U1-U4) and four items that assess intangible barriers (U5-U8) to being able to prepare healthful meals from the food a household has access to. Participants who selected “Sometimes true” or “Often true” were scored 1 for the item and those who select “Never true” were scored 0 for the item. Item scores were summed to create a 0-8 score. Higher scores are considered less desirable.

Households that score higher lack tangible components (e.g., food preparation equipment or sanitary areas) and/or face intangible issues (e.g., cooking skills and time) to prepare healthful meals. In a large sample of racially/ethnically diverse and largely low-income and food-insecure households across five states (CA, FL, MD, NC, and WA), “High” scores were 3.0 or above (the sample median was 2.0). Higher scores were associated with being food insecure and consuming “processed/heat-and-serve” meals more frequently and have poorer reported health and less frequent consumption of fruits and vegetables and “scratch-cooked” meals.

In addition to determining “low” and “high” scores, there may also be a need to understand how “high” scores could be improved. This measure offers practical information, particularly by comparing sub-scale scores to see if households face more tangible or intangible barriers, and also by examining item responses to identify specific barriers most impacting households. This information can be used to inform intervention development, assistance referrals, or evaluation/monitoring of existing programs. For example, perhaps households within a housing development lack food preparation equipment and sanitary areas to prepare meals and report limited cooking skills. A community kitchen could be implemented to provide equipment and sanitary environment as well as provide a space for cooking classes.

The potential for test bias was examined by test mode (paper versus web-based), age, race, gender, and education. There was no test bias detected in the pilot study sample for the characteristics assessed.

A two-item screener version was created for measurement applications in situations and settings that limit the number of items that can be administered (e.g., intake/clinical screening). The items U4 and U7 were selected for the brief screener version. Those who selected “Sometimes true” or “Often true” to either U4 **or** U7 screened positive for “high” Utilization Barriers. The two-item screener, compared to the full measure, had 96% sensitivity and 81% specificity for categorizing households as “high” on Utilization Barriers. Therefore, the households who screen positive are likely to need additional support relevant to the concepts assessed by the measure.

Table 4. Items comprising the Utilization Barriers measure along with item scoring and measure scoring. Tangible Barriers (U1-U4) and Intangible Barriers (U5-U8)

Item ID	Question Text	Response Options to All Items	Item Score	Measure Score
U1	In the last 12 months, (I/we) did not have access to a refrigerator, freezer, or other way to keep food from spoiling.	Never True = 0<br>Sometimes True = 1<br>Often True = 1		Sum of Item Scores
U2	In the last 12 months, (I/we) did not have a way to cook meals (e.g., stove, oven, microwave, hot plate or other appliance).			
U3	In the last 12 months, (I/we) did not have the kitchen tools or utensils needed to cook meals (e.g., pots, pans, a stirrer, can opener, knife, spoons/forks, or other utensils).			
U4	In the last 12 months, (I/we) did not have a clean and sanitary area to prepare meals.			
U5	In the last 12 months, (I/we) did not know how to select healthy foods from the food options (I/we) had.			
U6	In the last 12 months, (I/we) did not know how to make homemade meals from the food options (I/we) had (e.g., "meals from scratch" or meals without pre-made items).			
U7	In the last 12 months, (I/we) could not make a healthy meal from the food options (I/we) had.			
U8	In the last 12 months, (I/we) did not have time to cook meals.			

3.3. Food Insecurity Stability

Table 5 shows scoring for the three items for the Food Insecurity Stability measure. The scores to assess Food Insecurity Stability are calculated from three items that are follow-ups to three items in the USDA's household food security survey module, which are numbered HH2, HH3, and HH4. If a participant selects "Sometimes true" to HH2-HH4, they are then asked a follow-up question to clarify the timeframe that the statement is "Sometimes true" for their household. The options are (select all that apply): "Spring," "Summer," "Fall," "Winter," "Beginning of the month," "Middle of the month," "End of the month," and "Randomly, no certain timeframe." Selecting one or more seasons gives the participant a point for seasonal food insecurity, selecting one or more times of the month gives the participant a point for monthly food insecurity, and selecting "Randomly, no certain timeframe" gives the participant a point for intermittent food insecurity. If the participant selects "Often true" for HH2-HH4 they are not asked a follow-up question but are given a point for chronic food insecurity. Points are then summed, and each participant receives a score from 0-3 for chronic (C1-3), seasonal (S1-3), monthly (M1-3), and intermittent (I1-3) food insecurity.

Higher scores for the four stability types indicate a greater degree of experiencing chronic, seasonal, monthly, and intermittent food insecurity. In a large sample of racially/ethnically diverse and largely low-income and food-insecure households across five states (CA, FL, MD, NC, and WA), "High" scores were 1.0 or above (the sample median was 0.0). Higher scores were generally associated with being food insecure. Also, Chronic Food Insecurity was associated with consuming "processed/heat-and-serve" meals more frequently, and having poorer reported health and less frequent consumption of fruits and vegetables and "scratch-cooked" meals.

In addition to determining "low" and "high" scores, there may also be a need to understand how "high" scores could be improved. This measure offers practical information, particularly by allowing those who implement the measures to understand the temporal nature of households' food insecurity. Such information could be important in timing interventions and provision of resources.

The potential for test bias was examined by test mode (paper versus web-based), age, race, gender, and education. There was no test bias detected in the pilot study sample for the characteristics assessed.

Table 5. Approach to converting responses to the items within the Absorptive Capacity measure into a score for the full measure.

USDA Household Food Security Module Questions		Food Insecurity Stability Questions and Response Options		Item Scoring	Sum Stability Scores[A]
HH2. "(I/We) worried whether (my/our) food would run out before (I/we) got money to buy more."	Never True			0	
	Sometimes True	[C1; S1; M1; I1] In the last 12 months, when were you usually worried about running out of food? (Select all that apply)	Spring, Summer, Fall, and/or Winter	1	Add to Seasonal Stability Score
			Beginning of the month, Middle of the month, and/or End of the month	1	Add to Monthly Stability Score
			Randomly, no certain timeframe	1	Add to Intermittent Stability Score
	Often True			1	Add to Chronic Stability Score
HH3. "The food that (I/we) bought just didn't last, and (I/we) didn't have money to get more."	Never True			0	
	Sometimes True	[C2; S2; M2; I2] In the last 12 months, when did your household usually run out of food before getting money to buy more? (Select all that apply)	Spring, Summer, Fall, and/or Winter	1	Add to Seasonal Stability Score
			Beginning of the month, Middle of the month, and/or End of the month	1	Add to Monthly Stability Score
			Randomly, no certain timeframe	1	Add to Intermittent Stability Score
	Often True			1	Add to Chronic Stability Score
HH4. "(I/we) couldn't afford to eat balanced meals."	Never True			0	
	Sometimes True	[C3; S3; M3; I3] In the last 12 months, when was your household not able to afford to eat balanced meals? (Select all that apply)	Spring, Summer, Fall, and/or Winter	1	Add to Seasonal Stability Score
			Beginning of the month, Middle of the month, and/or End of the month	1	Add to Monthly Stability Score
			Randomly, no certain timeframe	1	Add to Intermittent Stability Score
	Often True			1	Add to Chronic Stability Score
[A]Sum the Chronic, Seasonal, Monthly, and Intermittent Scores separately, each can range from 0 to a maximum of 3.					

Appendix

Supplementary Table. Items and response options for the Availability, Utilization, and Stability measures.

Measure	Sub-scale/ Sub-topic	Item Number From Testing	Item Name	Item	Response Options
Perceived Limited Availability	Note: These questions are used to describe household food acquisition and to inform skip logic for AvS1-3 and AvP1-3. These are not part of the score.	A	Food Store Locations	Part A. In the last 12 months, from which of the following food stores has your household gotten food?<br>(Select all that apply)	Supermarket or grocery store (mostly sells food and household items) - Discount or big box store like Target or Walmart - Wholesale club like Costco, B.J.'s, or Sam's Club - Dollar store, 99 cent store, or similar place - Convenience store (e.g., 7-11 or MiniMart), corner store, or another similar place - Farmer's market - Produce store or fruit and vegetable stand - Restaurant, cafeteria, fast food, or another similar place - None of the above - Don't know
		B	Free Food Locations	Part B. In the last 12 months, from which of the following sources has your household gotten food?<br>(Select all that apply)	Food banks, food pantries, religious sites, 'Meals on Wheels,' or other places or programs that offer free food - Food donated from friends, family, neighbors, or other people you know - Food we grow or harvest, and/or we go hunting/ fishing for food - Found discarded food to eat - None of the above - Don't know
	Limited Food Store Availability	AvS1	Fruits and vegetables at stores	In the last 12 months, the food stores (I/we) went to had very few quality fruits and vegetables.	Never true - Sometimes true - Often true - Don't know
		AvS2	Foods we like at stores	In the last 12 months, the food stores (I/we) went to had very few foods that (I/we) liked.	Never true - Sometimes true - Often true - Don't know
		AvS3	Healthful food at stores	In the last 12 months, the food stores (I/we) went to had very few foods that were good for (my/our) health and well-being.	Never true - Sometimes true - Often true - Don't know
	Limited Food Pantry Availability (Note: Only asked of households that utilize food pantries)	AvP1	Fruits and vegetables at food pantries	In the last 12 months, the places (I/we) got free food had very few quality fruits and vegetables.	Never true - Sometimes true - Often true - Don't know
		AvP2	Foods we like at food pantries	In the last 12 months, the places (I/we) got free food had very few foods that (I/we) liked.	Never true - Sometimes true - Often true - Don't know
		AvP3	Healthful food at food pantries	In the last 12 months, the places (I/we) got free food had very few foods that met (my/our) religious or cultural needs.	Never true - Sometimes true - Often true - Don't know

Measure	Sub-scale/ Sub-topic	Item Number From Testing	Item Name	Item	Response Options
Utilization Barriers	Tangible Barriers	U1	Safe storage	In the last 12 months, (I/we) did not have access to a refrigerator, freezer, or other way to keep food from spoiling.	Never true - Sometimes true - Often true - Don't know
		U2	Cooking equipment	In the last 12 months, (I/we) did not have a way to cook meals (e.g., stove, oven, microwave, hot plate or other appliance).	Never true - Sometimes true - Often true - Don't know
		U3	Food preparation utensils	In the last 12 months, (I/we) did not have the kitchen tools or utensils needed to cook meals (e.g., pots, pans, a stirrer, can opener, knife, spoons/forks, or other utensils).	Never true - Sometimes true - Often true - Don't know
		U4	Sanitary cooking space	In the last 12 months, (I/we) did not have a clean and sanitary area to prepare meals.	Never true - Sometimes true - Often true - Don't know
	Intangible Barriers	U5	Select healthy foods	In the last 12 months, (I/we) did not know how to select healthy foods from the food options (I/we) had.	Never true - Sometimes true - Often true - Don't know
		U6	Scratch cooking skills	In the last 12 months, (I/we) did not know how to make homemade meals from the food options (I/we) had (e.g., "meals from scratch" or meals without pre-made items).	Never true - Sometimes true - Often true - Don't know
		U7	Healthy cooking	In the last 12 months, (I/we) could not make a healthy meal from the food options (I/we) had.	Never true - Sometimes true - Often true - Don't know
		U8	Cooking time	In the last 12 months, (I/we) did not have time to cook meals.	Never true - Sometimes true - Often true - Don't know
Food Insecurity Stability[A]	Food Insecurity Stability[A]	C1; S1; M1; I1	Follow-up to HH2	In the last 12 months, when were you usually worried about running out of food? (Select all that apply)	Spring - Summer - Fall - Winter - Beginning of the month - Middle of the month - End of the month - Randomly, no certain time frame - Don't know
		C2; S2; M2; I2	Follow-up to HH3	In the last 12 months, when did your household usually run out of food before getting money to buy more? (Select all that apply)	Spring - Summer - Fall - Winter - Beginning of the month - Middle of the month - End of the month - Randomly, no certain time frame - Don't know
		C3; S3; M3; I3	Follow-up to HH4	In the last 12 months, when was your household not able to afford to eat balanced meals? (Select all that apply)	Spring - Summer - Fall - Winter - Beginning of the month - Middle of the month - End of the month - Randomly, no certain time frame - Don't know

[A]These must be used in conjunction with HH2, HH3, and HH4 from USDA's Household Food Security Survey Module.
